# Evolution of *RAS* Mutations in Cell-Free DNA of Patients with Tissue *RAS* Wild-Type Metastatic Colorectal Cancer Receiving First-Line Treatment: The PERSEIDA Study

**DOI:** 10.3390/cancers14246075

**Published:** 2022-12-09

**Authors:** Manuel Valladares-Ayerbes, Pilar Garcia-Alfonso, Jorge Muñoz Luengo, Paola Patricia Pimentel Caceres, Oscar Alfredo Castillo Trujillo, Rosario Vidal-Tocino, Marta Llanos, Beatriz Llorente Ayala, Maria Luisa Limon Miron, Antonieta Salud, Luis Cirera Nogueras, Rocio Garcia-Carbonero, Maria Jose Safont, Esther Falco Ferrer, Jorge Aparicio, Maria Angeles Vicente Conesa, Carmen Guillén-Ponce, Paula Garcia-Teijido, Maria Begoña Medina Magan, Isabel Busquier, Mercedes Salgado, Ariadna Lloansí Vila

**Affiliations:** 1 Hospital Universitario Virgen del Rocío, 41013 Sevilla, Spain; 2 Hospital General Universitario Gregorio Marañón, 28007 Madrid, Spain; 3 Hospital San Pedro de Alcántara, 10003 Cáceres, Spain; 4 Complejo Hospitalario Area II de Cartagena, Hospital Universitario Santa Lucia, 30202 Cartagena, Spain; 5 Hospital Universitario Central de Asturias, ISPA, 33011 Oviedo, Spain; 6 Complejo Asistencial Universitario de Salamanca, IBSAL, 37007 Salamanca, Spain; 7 Hospital Universitario de Canarias, 38320 San Cristóbal de La Laguna, Spain; 8 Hospital Universitario de Burgos, 09006 Burgos, Spain; 9 Hospital Universitario Arnau de Vilanova, 25198 Lleida, Spain; 10 Hospital Mutua de Terrassa, 08221 Terrassa, Spain; 11 Hospital Universitario 12 de Octubre, Imas12, UCM, 28041 Madrid, Spain; 12 Hospital General Universitario de Valencia, 46014 València, Spain; 13 Hospital Universitari Son Llàtzer, 07198 Palma, Spain; 14 Hospital Universitari i Politècnic La Fe, 46026 València, Spain; 15 Hospital General Universitario José Maria Morales Meseguer, 30008 Murcia, Spain; 16 Hospital Universitario Ramón y Cajal, IRYCIS, 28034 Madrid, Spain; 17 Hospital Universitario San Agustín, 33401 Avilés, Spain; 18 Hospital Universitario Torrecárdenas, 04009 Almería, Spain; 19 Consorcio Hospitalario Provincial de Castellón, 12002 Castellón de la Plana, Spain; 20 Complexo Hospitalario de Ourense, 32005 Ourense, Spain; 21 Amgen S.A., 08039 Barcelona, Spain

**Keywords:** colorectal cancer, cell-free DNA, *RAS* mutations, solid biopsy

## Abstract

**Simple Summary:**

Cell-free DNA *RAS* mutation is being increasingly monitored in metastatic colorectal cancer (mCRC) for disease molecular characterization and selecting eligible patients for anti-EGFR initiation and rechallenge. Here, we monitored a homogeneous mCRC *RAS* wild-type (as per baseline solid biopsy) population starting first-line treatment using a BEAMing technique at three different mutant allele fraction (MAF) sensitivity cut-offs and we characterized the role of each MAF threshold and its correlation with clinical variables.

**Abstract:**

The serial analysis of cell-free DNA (cfDNA) enables minimally invasive monitoring of tumor evolution, providing continuous genetic information. PERSEIDA was an observational, prospective study assessing the cfDNA *RAS* (*KRAS*/*NRAS*) mutational status evolution in first-line, metastatic CRC, *RAS* wild-type (according to baseline tumor tissue biopsy) patients. Plasma samples were collected before first-line treatment, after 20 ± 2 weeks, and at disease progression. One hundred and nineteen patients were included (102 received panitumumab and chemotherapy as first-line treatment—panitumumab subpopulation). Fifteen (12.6%) patients presented baseline cfDNA *RAS* mutations (*n* = 14 [13.7%], panitumumab subpopulation) (mutant allele fraction ≥0.02 for all results). No patients presented emergent mutations (cfDNA *RAS* mutations not present at baseline) at 20 weeks. At disease progression, 11 patients (*n* = 9; panitumumab subpopulation) presented emergent mutations (*RAS* conversion rate: 19.0% [11/58]; 17.7% [9/51], panitumumab subpopulation). In contrast, three (5.2%) patients presenting baseline cfDNA *RAS* mutations were *RAS* wild-type at disease progression. No significant associations were observed between overall response rate or progression-free survival and cfDNA *RAS* mutational status in the total panitumumab subpopulation. Although, in patients with left-sided tumors, a significantly longer progression-free survival was observed in cfDNA *RAS* wild-type patients compared to those presenting cfDNA *RAS* mutations at any time. Continuous evaluation of *RAS* mutations may provide valuable insights on tumor molecular dynamics that can help clinical practice.

## 1. Introduction

Colorectal cancer (CRC) is the third most diagnosed cancer in men and the second most diagnosed cancer in women, and it is the second most common cause of cancer death in men and the third most common cause in women globally [1]. In Europe, the incidence in 2020 was 150,000 and 191,000 new cases in women and men, respectively [2], whereas in Spain, over 43,370 new cases were predicted for 2022 in the total population [3]. The recent advances in cytotoxic chemotherapy and targeted agents have improved overall survival, doubling it over the last 20 years up to 30 months [4].

In patients with metastatic CRC (mCRC), *RAS* (*KRAS*/*NRAS*) mutational status currently guides the therapeutic use of epidermal growth factor receptor (EGFR) inhibitors [4]. Tumor tissue biopsy testing is the standard of care to assess *RAS* (*KRAS*/*NRAS*) mutation in these patients [5,6,7,8]. Its determination must be performed in the primary tumor or the metastatic tissue upon the diagnosis of metastatic disease according to current treatment guidelines [4].

Detection of genetic alterations in tumor DNA is increasingly used for diagnostic, prognostic, and treatment purposes. Usually, genetic alterations are detected from archived tumor samples collected at a specific time, which do not provide information on disease progression or heterogeneity. Colorectal cancer harbors a considerable heterogenicity, with temporal and spatial differences in genetic mutations [9]. Tumor cells release 150–~200 base pair fragments of circulating tumor DNA (ctDNA) into the bloodstream [10]. This ctDNA normal half-life is less than an hour and contains the same genetic and epigenetic characteristics and mutations of the tumor [10], providing key information related to tumor development, progression, and resistance to treatment. The analysis of plasma ctDNA, also called “liquid biopsy”, has been actively studied and tested as a possible alternative to the invasive techniques for obtaining tumor insight. Liquid biopsy enables minimally invasive monitoring of tumor evolution which could provide continuous genetic information about the tumor while the patients is being treated and may overcome some of the challenges associated with tumor heterogeneity, such as the spatial and temporal heterogeneity [11,12,13].

Despite all of the liquid biopsy advantages, one of the main inconveniences is the sensitivity of the techniques used. ctDNA may vary between 0.01% and 93% of the total circulating free DNA (cfDNA) [14,15]. Some studies to detect *KRAS* mutations in cfDNA by PCR have not provided adequate concordance with the results obtained from solid biopsies [16,17,18]. Thus, alternative techniques such as BEAMing, which are highly sensitive for detecting the presence of point mutations in cfDNA even when they are uncommon, are required [19,20]. Aiming to provide new evidence on the potential added value of baseline liquid biopsy genotyping in the first-line setting, the primary objective of this observational, prospective study was to assess the concordance of the *RAS* (*KRAS*/*NRAS*) mutational status assessed in tissue samples and plasma samples (using the BEAMing technique) at baseline in *RAS* wild-type mCRC patients starting their standard first-line treatment. Additionally, the *RAS* (*KRAS*/*NRAS*) mutational status was assessed at 20 weeks after treatment initiation and at disease progression to first-line treatment. Moreover, the associations between *RAS* mutation status and different outcomes according to different mutant allele fraction (MAF) cut-offs and the predictive factors of progression-free survival (PFS) and tumor burden were explored in the panitumumab-treated subpopulation.

## 2. Materials and Methods

PERSEIDA (NCT02792478) was a nationwide, observational, multi-center, prospective study designed to evaluate the *RAS* (*KRAS*/*NRAS*) mutational status in liquid biopsies in first-line, mCRC, *RAS* wild-type (according to baseline tumor tissue biopsy) patients. The participants were managed following standard clinical practice, including the selection of the first-line treatment and the baseline tumor tissue biopsy.

Blood samples were collected before starting first-line treatment (baseline), at 20 ± 2 weeks after starting the treatment (prior to the second radiological assessment of the tumor response) and at disease progression coinciding with routine blood withdrawals. The samples were centrifuged to obtain plasma. The plasma was then frozen and maintained at −80 °C until it was shipped to Sysmex Inostics GmbH (Hamburg, Germany) for BEAMing analysis [21] at the end of the study. Accordingly, all of the investigators were blind to the BEAMing results during the study. The 20 weeks after starting treatment timepoint was selected following the Diaz et al. estimations of mutant *KRAS* fragments predicted to become evident after anti-EGFR initiation [22].

Tumor response was evaluated approximately every 3 months following RECIST version 1.1 criteria [23] until tumor progression, following clinical practice.

The inclusion criteria were: patients ≥18 years, with mCRC measurable by RECIST, who have started first-line treatment and with a histologically confirmed diagnosis of mCRC and wild-type *RAS* (according to baseline tumor tissue biopsy). The exclusion criteria were: pregnant or breastfeeding women, patients who have previously received monoclonal antibodies against EGFR (cetuximab or panitumumab), small-molecule EGFR inhibitors (such as erlotinib) or other biological cancer treatments, patients with a history of another solid or hematological tumor in the previous 5 years (except a history of basal cell carcinoma of the skin or pre-invasive cervical cancer), and patients who were participating or had participated in a clinical trial in the 30 days prior to inclusion.

The protocol was approved by an independent ethics committee, and all of the patients gave their written informed consent before enrollment.

The patients were recruited consecutively and were followed-up until disease progression. At baseline, the following variables were collected: demographic data, relevant medical history (including CRC-related data: date of histological diagnosis, primary location, previous surgery and outcome, previous treatments, adjuvant or neoadjuvant intention, and affected organs), physical examination, tumor lesions according to RECIST criteria, ECOG performance status, first-line treatment, and laboratory parameters (hematologic and biochemical data and serum carcinoembryonic antigen concentrations). Tumor assessment and safety data were collected during the treatment, at the end of first-line treatment, and at follow-up visits.

### Statistical Analysis

The primary endpoint was the detection rate of *RAS* (*KRAS*/*NRAS*) mutations in liquid biopsies at baseline. Secondary endpoints included description of *RAS* (*KRAS*/*NRAS*) mutations in liquid biopsies at disease progression and at 20 ± 2 weeks. The detection rate was defined as the percentage of patients who had *RAS* mutations in liquid biopsies in patients with wild-type *RAS* according to solid biopsy (percentage of discordant patients, together with its 95% confidence interval [CI]). This detection rate was calculated considering three different MAF cut-off points (≥1%, ≥0.1% and ≥0.02%). The percentages of patients with mutations on different *KRAS* and *NRAS* exons (*KRAS*: exon 2, codons 12 [mutations: c.34G>A, c.34G>C, c.34G>T, c.35G>A, c.35G>C, c.35G>T] and 13 [c.38G>A]; exon 3, codon 61 [c.182A>T, c.183A>C, c.183A>T]; exon 4, codon 146 [c.436G>A]; NRAS: exon 2, codons 12 [c.35G>A] and 13 [c.38G>A]; exon 3, codon 61 [c.181C>A, c.182A>G, c.182A>T, c.183A>T, c.183A>C]) were also obtained.

Moreover, the conversion rate at disease progression was calculated, defined as the percentage of patients who had *RAS* wild-type status at baseline (in solid and liquid biopsy) that converted to *RAS* mutated in liquid biopsy at disease progression (emergent mutations).

In addition, the association between cfDNA *RAS* mutational status and overall response rate (ORR), PFS, and overall survival (OS) was explored in the panitumumab-treated subpopulation. PFS was defined as the time from the start date of treatment until objective tumor progression, initiation of second-line treatment, or all-cause death, whichever occurred first. Progression was derived from the response according to RECIST version 1.1 criteria [23]. OS was defined as the time from the start date of treatment until all-cause death. ORR was defined as the proportion of patients who had a partial (PR) or complete response (CR) to therapy according to the RECIST criteria, not including stable disease (SD). The best overall response for CR and PR was considered confirmed if assessed in at least two consecutive evaluations, performed no less than 28 days after the response criteria was met for the first time. SD required an SD response or better at a visit at least 49 days after the start of treatment.

PFS and OS analyses were performed using the Kaplan–Meier method, and a multivariable Cox regression analysis was also used to explore the predictive factors of PFS (providing hazard ratios [HR] and 95% CIs). The following variables were included in the univariable model: *RAS* status at any time by MAF cut-off (wild-type/mutant), number of affected organs (1/>1), ECOG performance status (0/>0), age, primary tumor location (left/right colon), primary tumor surgery (yes/no), number of metastasis localizations, and Köhne prognostic score (high/intermediate/low risk). Those variables with *p*-value < 0.15 at univariable model were included into the multivariable model, along with *RAS* status at any time (MAF ≥ 0.02%). Furthermore, a multivariable linear regression model was used to explore the predictive factors of tumor burden.

Changes in continuous variables over time were analyzed using paired *t*-tests. Differences between subgroups of patients were tested using Student’s *t*-tests, Mann–Whitney tests, or Chi-squared tests, as applicable. Descriptive analyses were provided for each variable at all the study visits.

Statistical analyses were performed with the SAS statistical software package (SAS Institute, Inc., Cary, NC, USA).

## 3. Results

### 3.1. Baseline Characteristics

One hundred and twenty-nine (*n* = 129) patients were screened in 25 Spanish hospitals between May 2016 and March 2020, of which 119 were included (evaluable population). In the evaluable population, 113 patients received chemotherapy plus anti-EGFR, 4 patients received chemotherapy plus anti-VEGF, and 2 patients received chemotherapy alone. The most frequently initiated first-line treatment was panitumumab plus chemotherapy (*n* = 102), constituting the panitumumab subpopulation. Regarding chemotherapy, the most frequently initiated regimen was FOLFOX in 94 patients (in combination with panitumumab [*n* = 85], cetuximab [*n* = 8] or alone [*n* = 1]) followed by FOLFIRI in 14 patients (in combination with panitumumab [*n* = 11] or cetuximab [*n* = 3]). Table 1 displays the main demographic and clinical characteristics of both populations. Most patients were male, with a mean age of 62 years and an ECOG of 0 or 1. The mean time since CRC diagnosis was 6 months, with 80% of patients presenting left tumor location and 37% of patients presenting at least one previous CRC surgery. The mean time between *RAS* determination in solid biopsy and the baseline liquid biopsy was 1.03 months in the evaluable population and 1.17 months in the panitumumab subpopulation.

### 3.2. Primary Endpoint

A total of 15 (12.6%) patients presented *RAS* (*KRAS*/*NRAS*) mutations (MAF ≥ 0.02) in liquid biopsies at baseline in the evaluable population (*n* = 14 [13.7%] in the panitumumab subpopulation), with decreased rates at higher MAF cut-offs (Table 2). Accordingly, the percentage of *RAS* mutational status concordance between solid and liquid biopsies was 87.4% in the evaluable population and 86.3% in the panitumumab subpopulation at baseline (MAF ≥ 0.02) (Table 2). A logistic regression analysis did not find any variables associated with discordant cases at baseline (data not shown).

### 3.3. Secondary Endpoints

**Figure 1:**
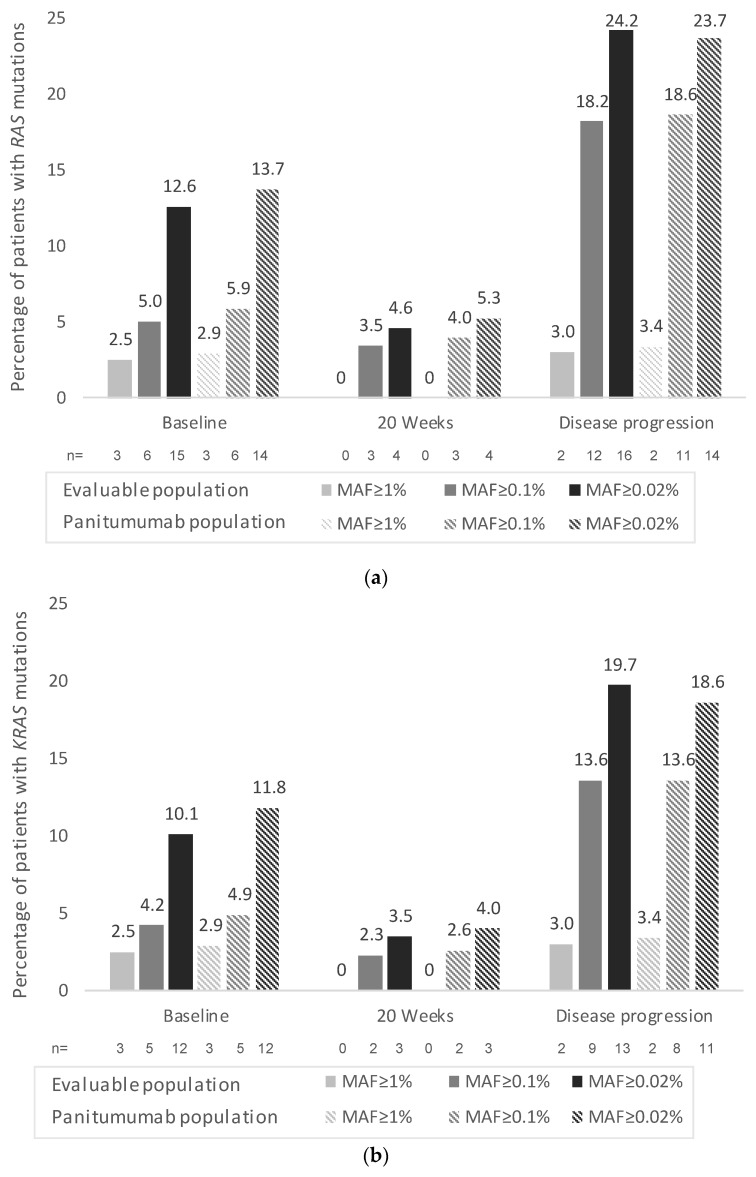

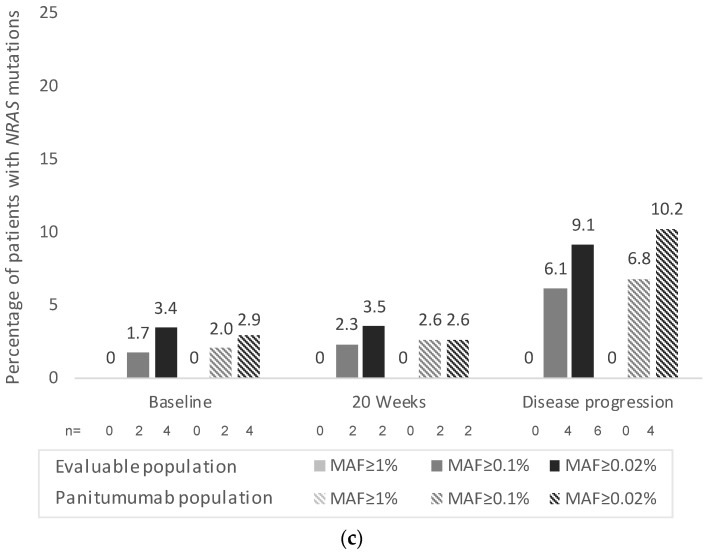
Percentage of patients with (**a**) *RAS,* (**b**) *KRAS,* and (**c**) *NRAS* mutations in liquid biopsies at baseline, at 20 weeks (±2 weeks), and at disease progression according to mutant allele fraction (MAF) cut-offs. One (*n* = 1) patient had both *KRAS* and *NRAS* mutations (MAF ≥ 0.1% and MAF ≥ 0.02%). n: number of patients with mutations. Percentages calculated based on patients with available samples.

A total of 4 (4.6%) patients presented cfDNA *RAS* (*KRAS*/*NRAS*) mutations at 20 weeks and 16 (24.2%) patients presented cfDNA *RAS* (*KRAS*/*NRAS*) mutations at disease progression (both percentages calculated at MAF ≥ 0.02) in the evaluable population (Figure 1A). The percentages of patients with *RAS* (*KRAS* and *NRAS*) mutations in liquid biopsies at 20 weeks and at disease progression according to the different MAF cut-offs in the evaluable population and panitumumab subpopulation are shown in Figure 1.

At disease progression, a total of 11 patients (*n* = 9 in the panitumumab subpopulation) presented *RAS* mutations in liquid biopsies (MAF ≥ 0.02%) that were not present at baseline (by solid and liquid biopsy). Accordingly, the *RAS* conversion rate (emergent mutations) at disease progression was 19.0% (11/58 patients with baseline *RAS* wild-type status [by solid and liquid biopsy] and blood samples available at disease progression) in the evaluable population and 17.7% (9/51 patients) in the panitumumab subpopulation (MAF ≥ 0.02%). The conversion rates according to the different MAF cut-offs are displayed in Table 2 for both populations. As expected, the conversion rates were higher at lower MAF cut-offs. No patients presented *RAS* mutations in liquid biopsies at 20 weeks that were not present at baseline (0% conversion rate at 20 weeks).

The characteristics of patients with *RAS* mutations at any time (per liquid biopsy), including codon–exon–amino acid position/change, primary tumor location, site of metastasis, first-line treatment, best overall response, and PFS are shown in Supplementary Table S1. Ten (*n* = 10) out of the eleven patients presenting *RAS* mutations in liquid biopsies (MAF ≥ 0.02%) at disease progression that were not present at baseline achieved PR as the best overall response, while one achieved SD. In these patients, the most frequent metastatic site was the liver (*n* = 8). Moreover, three (5.2%) patients presenting *RAS* mutations in liquid biopsies (MAF ≥ 0.02%) at baseline were *RAS* wild-type at disease progression (Supplementary Table S1, patients #1, #2 and #5). These three patients reached PR or CR, received FOLFOX plus panitumumab, and had liver or liver plus lung metastases.

### 3.4. Exploratory Endpoints (Only Assessed in the Panitumumab Subpopulation)

A total of 93 patients had data available for the response (not confirmed) in the panitumumab subpopulation. The ORR was 75.3% (*n* = 70; CR: 18.3% [*n* = 17], PR: 57.0% [*n* = 53]) in the total panitumumab subpopulation, with the rate being 78.1% (57/73) and 65.0% (13/20) in patients with left- and right-sided primary tumor location, respectively.

The ORR according to *RAS* (*KRAS*/*NRAS*) mutational status at baseline and at any time and by tumor location is shown in Table 3. Considering the *RAS* status at baseline, the ORR was numerically higher in the left-sided, *RAS* wild-type tumors compared to the right-sided (all right-sided tumors were *RAS* wild-type) and the *RAS* mutant tumors (in all of the different MAF cut-offs, not significative). Similar results were observed when considering the *RAS* mutational status at any time. Among patients with right-sided tumors, there were only one (MAF ≥ 0.1%) and two patients (MAF ≥ 0.02%) with *RAS* mutant status (both achieving ORR), thus preventing direct comparisons (Table 3). A tendency to increased ORR rates was observed at diminishing MAF cut-offs in *RAS* mutant patients at baseline and at any time.

**Figure 2:**
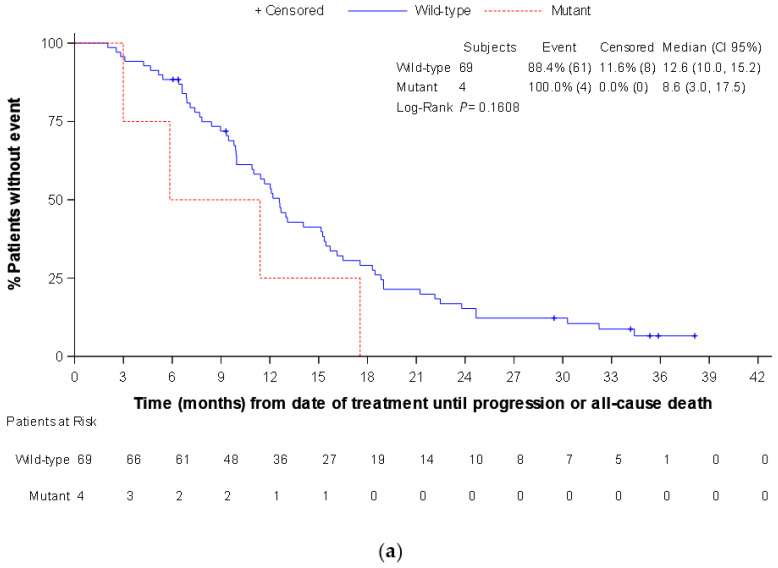

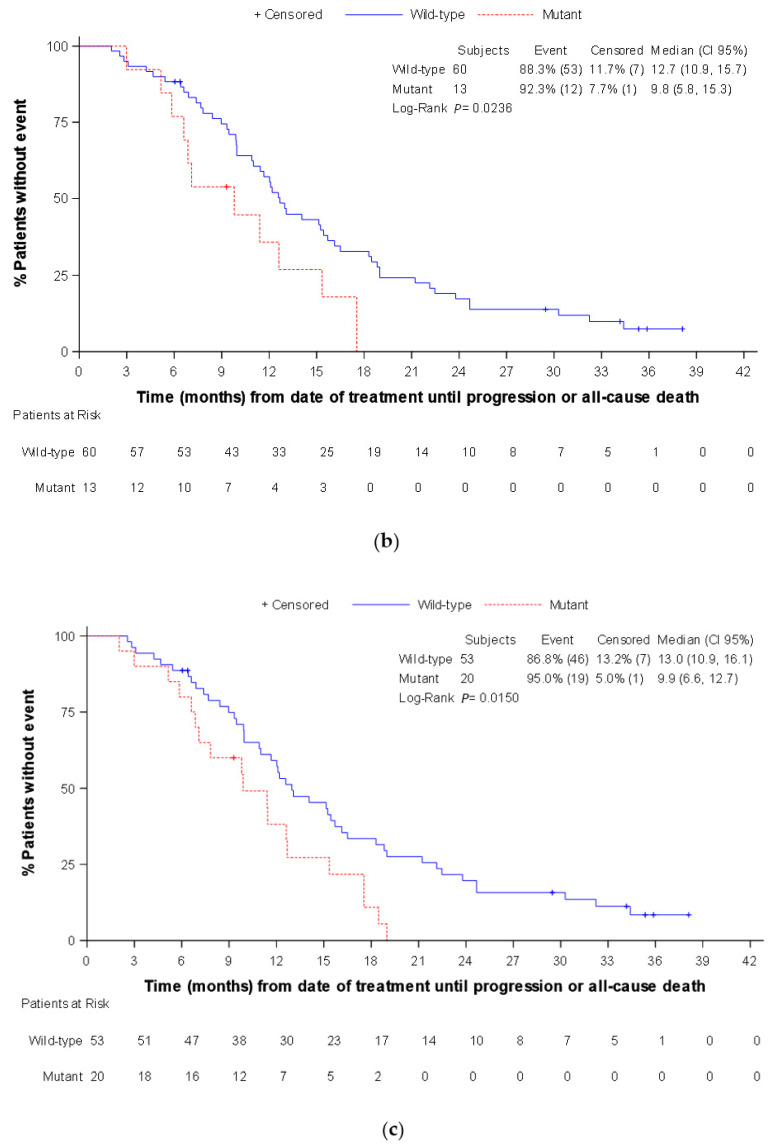
Progression free survival according to *RAS* mutational status in liquid biopsy at any time (panitumumab subpopulation, left tumor location): (**a**) mutant allele fraction ≥1% cut-off; (**b**) mutant allele fraction ≥0.1% cut-off; and (**c**) mutant allele fraction ≥0.02% cut-off.

Regarding PFS, the median (CI 95%) time in the total panitumumab subpopulation was 12.1 (10.0–13.8) months. There were no statistically significant differences in PFS between patients with *RAS* wild-type and *RAS* mutated in the different MAF cut-offs, both at baseline and at any time (data not shown). Similar results were observed by *RAS* status at baseline in the subgroup of patients with left-sided tumors. By contrast, in this subgroup there were statistically significant differences in the median PFS between patients with *RAS* wild-type and *RAS* mutated at any time in MAF ≥ 0.02% cut-off (13.0 [IC 95%: 10.9–16.1] vs. 9.9 [6.6–12.7] months, respectively; *p* = 0.015), and MAF ≥ 0.1% cut-off (12.7 [IC 95%: 10.9–15.7] vs. 9.8 [5.9–15.3] months, respectively; *p* = 0.024) (Figure 2).

Finally, the median OS in the total panitumumab subpopulation was not reached, with a total of 11 events. There were no statistically significant differences in OS between patients with *RAS* wild-type and *RAS* mutated at any time in the MAF ≥ 0.1% and ≥0.02% cut-offs, while it was observed in the MAF ≥ 1%, where the four patients with *RAS* mutated had a median OS of 17.4 months, while it was not reached in the *RAS* wild-type patients (*p* = 0.01) (data not shown).

The multivariable Cox regression model for PFS did not yield any statistically significant results, although a tendency towards an increased probability of PD was observed in *RAS* mutated patients at any time (MAF > 0.02%) compared to *RAS* wild-type patients (HR: 1.54 [95% CI: 0.93–2.56]; *p* = 0.096) (Supplementary Table S2).

Regarding tumor burden, the multivariable linear regression model showed that the difference in the estimated means (sum of the longest diameters) between patients with and without liver metastasis was 29.6 mm (95% CI: 1.02–58.3; *p* = 0.043). Additionally, the cfDNA concentration was significantly associated with the tumor burden (*p* < 0.0001). In contrast, the presence of *RAS* mutations at baseline was not associated with tumor burden in our model (Supplementary Table S3).

We also performed exploratory, post-hoc logistic regressions to analyze the significance of MAF as a predictor and ROC curve analyses to estimate the best MAF cut-off point for predicting the clinical response, without achieving any significant results (data not shown).

## 4. Discussion

In our prospective, multicentric, observational study, we investigated the concordance of the *RAS* (*KRAS*/*NRAS*) mutational status between solid and liquid biopsies in patients with *RAS* wild-type mCRC (according to solid biopsy) at baseline managed following clinical practice. Our results showed a high concordance between tissue and plasma samples (ranging from 97% to 86% in the evaluable population and panitumumab subpopulation, according to MAF cut-off), with the concordance rate being similar to the rates reported in previous studies showing concordance rates between 86% and 93% [24,25,26,27]. It should be noted that previous similar studies comprised more heterogeneous populations compared to ours, including baseline *RAS* mutated (according to solid biopsy) patients in both first and subsequent lines of treatment. A recent publication by Kagawa, et al. [28] also using BEAMing analysis in mCRC patients (both *RAS* mutated and wild-type at baseline) with single-site metastasis suggests that the concordance rates may differ by metastatic site (91%, 88%, and 64% in patients with single metastases in the liver, peritoneum, and lung, respectively). Similar results were observed by Wang, et al. [29] using next-generation sequencing (NGS), reporting baseline *RAS* concordances of 90% and 37.5% in patients with only liver metastases and only lung metastases, respectively. Additionally, Kagawa, et al. reported the increased tumor burden (longest diameter and number of lesions) as the most significant factor associated with increased solid-liquid biopsies concordance [28]. More recent studies in heterogeneous populations have reported total number of lesions and total tumor burden as the most significant predictors of discordant cases [30,31,32]. In alingment, in our study the presence of liver metastasis was associated with an increased tumor burden, and the majority of patients presented liver metastasis, which may explain the high concordance rates observed.

Despite the high concordance between biopsies at baseline, we observed a *RAS* conversion rate at disease progression (emergent mutations) of 19% in the evaluable population and 17.7% in the panitumumab subpopulation, while there were three patients (5.2%) where the *RAS* mutations detected at baseline were not detected at disease progression. Previous studies have reported the results of emergent mutations in subsequent-lines of treatment [27,33,34,35]. However, little is known about the emergent mutations after first-line treatment. Recently, Parseghian, et al. [36] reported that acquired mutations in *KRAS*, *NRAS*, *BRAF*, *MAP2K1*, or *EGFR* rarely develop after first-line treatment (6.8%), contrary to what has been observed in second- and third-line treatment (40-50%). By contrast, Wang, et al. [29] reported 44.4% (4/9) of patients with baseline *RAS* mutations showed *RAS* clearance at disease progression after first-line treatment, while 27.3% (3/11) of patients showed new *RAS* mutations. Only 5/20 patients were treated in first line with anti-EGFR treatments. The increased frequency of emergent mutations at disease progression (secondary resistance) has been postulated to be attributable to the clonal evolution under the selective pressure of EGFR inhibition [37]. However, in the first-line setting, pre-existing subclonal mutations do not appear to be the dominant source of emergent mutations at disease progression, suggesting that there may also be a transient mutational process driving anti-EGFR resistance [36]. Similarly, other authors have also proposed that other mechanisms beyond *RAS* emergent mutations will probably play an important role, which may include alterations involved in chemotherapy and/or anti-EGFR intrinsic or/and acquired resistance [29,37]. A greater knowledge of the molecular complexity that develops as a result of EGFR inhibition will help to guide new strategies in refractory patients with mCRC [38].

In our study, we did not observe a significant association between ORR or PFS and *RAS* mutational status by liquid biopsies at baseline or at any time across MAF cut-offs in the total panitumumab subpopulation. It should be noted that the proportion of *RAS* mutant patients at baseline was small. However, the ORR and PFS results in these patients tended to improve as the MAF threshold decreased. Regarding OS, a statistically significant difference between patients with *RAS* wild-type and *RAS* mutated at any time was observed for the MAF ≥ 1%. These results should be interpreted with caution, since the median OS was not reached in the panitumumab subpopulation and the sample size of *RAS* mutated patients was very small (*n* = 4). Previously reported studies in second- and third-line panitumumab populations also found that the presence of emergent *RAS* mutations at disease progression was not associated with differences in PFS, ORR, or OS, despite their larger proportions of emergent *RAS* mutations [27,33,34,35].

Interestingly, when the left-sided tumor patients were analyzed, the median PFS was significantly longer among cfDNA *RAS* wild-type patients when compared to those presenting cfDNA *RAS* mutations at any time. This better prognosis observed in left-sided, *RAS* wild-type tumors is aligned with previously reported data [39,40,41], further highlighting the importance of primary tumor location and supporting the clinical differences between right- and left-sided colon tumors. Unfortunately, in our study no comparisons by tumor sidedness (left vs. right) were possible in patients with *RAS* mutant status due to the small sample size.

This study has some limitations. It was initially designed to determine the concordance of liquid and solid biopsies at baseline and the appearance of emergent *RAS* mutations up to disease progression, whereas the association of *RAS* mutational status and outcomes and the predictive factors study were only explorative endpoints. Furthermore, the study included small patient numbers to allow for strong evidence of some of the subpopulations analyses (e.g., right-sided, *RAS* mutant). Additionally, we only tested for mutations in *RAS* (*KRAS*/*NRAS*) in liquid biopsies, but the acquired resistance to anti-EGFR treatments in mCRC patients is known to be caused by several other mechanisms, including EGFR extracellular domain mutations, *MET* amplifications, *BRAF* mutations, and *HER2* amplifications [42]. Despite this, the results observed in this study are aligned with the previously reported data. Since this was an observational study, patients were treated following clinical practice, thus preventing us from drawing any conclusions on the role of specific treatment regimens on the results. However, in our study cfDNA *RAS* mutations by themselves did not predict a lack of clinical benefits to panitumumab plus chemotherapy. Additionally, there were three patients with *RAS* mutations at baseline that were *RAS* wild-type at progression. The reason for this change (e.g., reversion to wild-type, the sensitivity cut-off of the liquid biopsy, the occurrence of false positives) is unknown. Finally, the clinically relevant *RAS* MAF cut-off in liquid biopsies is yet to be defined. A follow-up and serial cfDNA *RAS* analyses could help us to understand their clinical and biological significances. This may play an important role in the future development and administration of *KRAS* inhibitors, especially when tumor tissue is not available.

Our study had some key strengths. The studied population was homogeneous, with all of the patients being *RAS* (*KRAS*/*NRAS*) wild-type at baseline as per standard-of-care solid biopsy, starting their first-line treatment (mostly panitumumab plus chemotherapy). Additionally, we performed a dynamic monitorization of cfDNA using a highly sensitive technique (BEAMing), evaluating the *RAS* mutational status over time. In addition, the correlation between RECIST response and PFS and the *RAS* mutational status was assessed, differentiating by primary tumor side.

In this sense, our results contribute to a growing body of work supporting the use of cfDNA biomarkers to predict PFS in patients with mCRC receiving first-line chemotherapy treatment. Nevertheless, limited data are available on the role of liquid biopsy to predict the outcomes of patients clinically eligible for anti-EGFR-based upfront treatment [43].

## 5. Conclusions

In summary, the concordance rate between liquid and solid biopsy was very high at baseline, consistent with previous studies. At disease progression, there was a considerable percentage of patients with emerging *RAS* mutations (19%) that may be potentially attributable to the acquired resistance to anti-EGFR treatments. However, in our exploratory analyses, the *RAS* mutations detected were not associated with differences in clinical outcomes, except in patients with left-sided tumors. Clinical outcomes tended to improve as the MAF threshold decreased.

## Figures and Tables

**Table 1:** Baseline demographic and clinical characteristics.

	Panitumumab Subpopulation ^1^ (*n* = 102)	Evaluable Population (*n* = 119)
Male, n (%)	63 (61.8)	73 (61.3)
Age (years), mean (SD)	62.2 (10.6)	62.3 (10.6)
BMI (Kg/m^2^), mean (SD)	26.0 (4.0)	25.8 (4.3)
ECOG performance status, n (%)		
0	48 (47.1)	56 (47.1)
1	50 (49.0)	59 (49.6)
2	1 (1.0)	1 (0.84)
Not available	3 (2.9)	3 (2.5)
Köhne prognostic score, n (%)		
Low risk	44 (43.1)	50 (42.0)
Medium risk	45 (44.1)	55 (46.2)
High risk	9 (8.8)	10 (8.4)
Not available	4 (3.9)	4 (3.4)
Time (months) since histological diagnosis, mean (SD)	6.0 (10.4)	6.2 (11.1)
Primary tumor location, n (%)		
Left colon	82 (80.4)	95 (79.8)
Right colon	20 (19.6)	24 (20.2)
Previous surgeries for colorectal cancer, n (%)	37 (36.3)	45 (37.8)
Prior treatment for colorectal cancer, n (%)	17 (16.7)	20 (16.8)
Radiotherapy	1 (1.0)	1 (0.8)
Chemotherapy	11 (10.8)	12 (10.1)
Radiotherapy and chemotherapy	5 (4.9)	7 (5.9)
No prior treatment	84 (82.4)	98 (82.4)
Affected organs, n (%)		
Liver	68 (66.7)	60 (67.2)
Lung	39 (38.2)	42 (35.3)
Basal ganglia	28 (27.5)	35 (29.4)
Peritoneum	18 (17.7)	22 (18.5)
Adrenal	8 (7.8)	8 (6.7)
Bone	4 (3.9)	4 (3.4)
Other	20 (19.6)	26 (21.9)
Sum of diameters of target lesions (mm), mean (SD)	89.9 (75.1)	88.1 (71.8)
Serum carcinoembryonic antigen (ng/mL), median (Q1, Q3)	38.6 (7.6, 176.5)	33.8 (6.8, 170.8)
Lactate dehydrogenase, ULN, median (Q1, Q3)	326.5 (211.0, 498.0)	312.0 (207.0, 478.0)
Time (months) since *RAS* wild-type determination by solid biopsy, mean (SD)	1.03 (3.29)	1.17 (3.47)
Solid biopsy extraction localization, n (%)		
Primary	88 (86.3)	104 (87.4)
Metastasis	14 (13.7)	15 (12.6)

1. Evaluable population treated with chemotherapy + panitumumab. BMI: body mass index; Q1: 25th percentile; Q3: 75th percentile; SD: standard deviation; ULN: upper limit of normality.

**Table 2:** Percentage of RAS mutations in liquid biopsies at baseline and conversion rate at disease progression according to MAF cut-offs.

	Panitumumab Subpopulation ^1^ (*n* = 102)			Evaluable Population ^1^ (*n* = 119)		
	MAF ≥ 1%	MAF ≥ 0.1%	MAF ≥ 0.02%	MAF ≥ 1%	MAF ≥ 0.1%	MAF ≥ 0.02%
**At baseline**						
*RAS* mutant detection rate, % (95% CI) ^2^	2.9 (0.6–8.4)	5.9 (2.2–12.4)	13.7 (7.7–22.0)	2.5 (0.5–7.2)	5.0 (1.9–10.7)	12.6 (7.2–19.9)
Negative percent agreement (*RAS*), % (95% CI) ^3^	97.1 (91.6–99.4)	94.1 (87.6–97.8)	86.3 (78.0–92.3)	97.5 (92.8–99.5)	95.0 (89.4–98.1)	87.4 (80.1–92.8)
**At disease progression**						
Patients that converted to *RAS* mutant at progression, n (%) ^4^	1 (1.0)	9 (8.8)	9 (8.8)	1 (0.8)	10 (8.4)	11 (9.2)
Conversion rate, % (95% CI) ^5^	1.7 (0.04–9.2)	15.8 (7.5–27.9)	17.7 (8.4–30.9)	1.5 (0.04–8.3)	15.6 (7.8–26.9)	19.0 (9.9–31.4)

^1^ At baseline, one patient had both *KRAS* and *NRAS* mutations (MAF ≥ 0.1% and MAF ≥ 0.02%). At disease progression, one patient had both *KRAS* and *NRAS* mutations (MAF ≥ 0.1%) and three patients had both *KRAS* and *NRAS* mutations (MAF ≥ 0.02%). ^2^ Percentage of discordant patients. ^3^ Percentage of concordant patients in *RAS* wild-type patients according to solid biopsy. ^4^ Patients who initially had *RAS* wild-type status (by solid and liquid biopsy) that converted to *RAS* mutant at disease progression (liquid biopsy, any mutation). ^5^ Percentages calculated on patients with baseline *RAS* wild-type status (by solid and liquid biopsy) and blood sample available at disease progression (*n* = 58/57/51 in the panitumumab subpopulation and *n* = 65/64/58 in the evaluable population for MAF ≥ 1%/≥0.1%/≥0.02%, respectively). CI: confidence interval using the Clopper–Pearson exact method; MAF: mutant allele fraction.

**Table 3:** Overall response rate according to *RAS* mutational status in liquid biopsy at baseline and at any time (panitumumab subpopulation, classified by primary tumor location).

		*RAS* Wild-Type	*RAS* Mutant	Odds Ratio (95% CI)
**At baseline**				
**Total population** (*n* = 93)				
MAF ≥ 1%	ORR ^1^, % (95% CI)	76.7% (66.6–84.9%)	33.3% (0.8–90.6%)	6.6 (0.6–76.1)
	n/N ^2^	69/90	1/3	
MAF ≥ 0.1%	ORR ^1^, % (95% CI)	76.1% (65.9–84.6%)	60.0% (14.7–94.7%)	2.1 (0.3–13.6)
	n/N ^2^	67/88	3/5	
MAF ≥ 0.02%	ORR ^1^, % (95% CI)	77.5% (66.8–86.1%)	61.5% (31.6–86.1%)	2.2 (0.6–7.4)
	n/N ^2^	62/80	8/13	
**Left-sided tumors** (*n* = 73)				
MAF ≥ 1%	ORR ^1^, % (95% CI)	80.0% (68.7–88.6%)	33.3% (0.8–90.6%)	8.0 (0.7–94.7)
	n/N ^2^	56/70	1/3	
MAF ≥ 0.1%	ORR ^1^, % (95% CI)	79.4% (67.9–88.3%)	60.0% (14.7–94.7%)	2.6 (0.4–16.9)
	n/N ^2^	54/68	3/5	
MAF ≥ 0.02%	ORR ^1^, % (95% CI)	81.7% (69.6–90.5%)	61.5% (31.6–86.1%)	2.8 (0.8–10.2)
	n/N ^2^	49/60	8/13	
**Right-sided tumors** (*n* = 20)				
MAF ≥ 1%	ORR ^1^, % (95% CI)	65.0% (40.8–84.6%)	0%	-
	n/N ^2^	13/20	0/0	
MAF ≥ 0.1%	ORR ^1^, % (95% CI)	65.0% (40.8–84.6%)	0%	-
	n/N ^2^	13/20	0/0	
MAF ≥ 0.02%	ORR ^1^, % (95% CI)	65.0% (40.8–84.6%)	0%	-
	n/N ^2^	13/20	0/0	
**At any time**				
**Total population** (*n* = 93)				
MAF ≥ 1%	ORR ^1^, % (95% CI)	76.4% (66.2–84.8%)	50.0% (6.8–93.2%)	3.2 (0.4–24.4)
	n/N ^2^	68/89	2/4	
MAF ≥ 0.1%	ORR ^1^, % (95% CI)	74.7% (63.6–83.8%)	78.6% (49.2–95.3%)	0.8 (0.2–3.2)
	n/N ^2^	59/79	11/14	
MAF ≥ 0.02%	ORR ^1^, % (95% CI)	74.7% (62.9–84.2%)	77.3% (54.6–92.2%)	0.9 (0.3–2.7)
	n/N ^2^	53/71	17/22	
**Left-sided tumors** (*n* = 73)				
MAF ≥ 1%	ORR ^1^, % (95% CI)	79.7% (68.3–88.4%)	50.0% (6.8–93.2%)	3.9 (0.5–30.4)
	n/N ^2^	55/69	2/4	
MAF ≥ 0.1%	ORR ^1^, % (95% CI)	78.3% (65.8–87.9%)	76.9% (46.2–95.0%)	1.1 (0.3–4.5)
	n/N ^2^	47/60	10/13	
MAF ≥ 0.02%	ORR ^1^, % (95% CI)	79.6% (65.9–89.2%)	75.0% (50.9–91.3%)	1.3 (0.4–4.3)
	n/N ^2^	42/53	15/20	
**Right-sided tumors** (*n* = 20)				
MAF ≥ 1%	ORR ^1^, % (95% CI)	65.0% (40.8–84.6%)	0%	-
	n/N ^2^	13/20	0/0	
MAF ≥ 0.1%	ORR ^1^, % (95% CI)	63.2% (38.4–83.7%)	100% (2.5–100%)	-
	n/N ^2^	12/19	1/1	
MAF ≥ 0.02%	ORR ^1^, % (95% CI)	61.1% (35.8–82.7%)	100% (15.8–100%)	-
	n/N ^2^	11/18	2/2	

^1^ Not confirmed. A total of 93 patients had available response data. ^2^ n: number of patients with partial response and complete response; N: number of patients with available response data. CI: confidence interval using the Clopper–Pearson exact method; MAF: mutant allele fraction; ORR: overall response rate.

## Data Availability

The data presented in this study are available on request from the corresponding author.

## Supplementary Materials

The following supporting information can be downloaded at: https://www.mdpi.com/article/10.3390/cancers14246075/s1. Supplementary Table S1: Characteristics of patients with RAS mutations at any time as per liquid biopsy (codon–exon–amino acid position/change); Supplementary Table S2: Univariable and Multivariable Cox Regression Model for Progression Free Survival (Panitumumab Subpopulation); Supplementary Table S3: Univariable and Multivariable Linear Regression Model for Tumor Burden (Panitumumab Subpopulation).
