# Supplementary material for: Evolution of RAS Mutations in Cell-Free DNA of Patients with Tissue RAS Wild-Type Metastatic Colorectal Cancer Receiving First-Line Treatment: The PERSEIDA Study
Source: Cancers (Basel). 2022 Dec 9;14(24):6075. doi: 10.3390/cancers14246075 (PMC9776941; doi:10.3390/cancers14246075)
Supplement: Supplementary file 1 [file cancers-14-06075-s001.zip › cancers-2027236-Supplementary Materials -1.pdf]

cancers

Supplementary Materials: Evolution of *RAS* Mutations in Cell-Free DNA of Patients with Tissue *RAS* Wild-Type Metastatic Colorectal Cancer Receiving First-Line Treatment: The PERSEIDA Study

Manuel Valladares-Ayerbes, Pilar Garcia-Alfonso, Jorge Muñoz Luengo, Paola Patricia Pimentel Caceres, Oscar Alfredo Castillo Trujillo, Rosario Vidal-Tocino, Marta Llanos, Beatriz Llorente Ayala, Maria Luisa Limon Miron, Antonieta Salud, Luis Cirera Nogueras, Rocio Garcia-Carbonero, Maria Jose Safont, Esther Falco Ferrer, Jorge Aparicio, Maria Angeles Vicente Conesa, Carmen Guillén-Ponce, Paula Garcia-Teijido, Maria Begoña Medina Magan, Isabel Busquier, Mercedes Salgado, Ariadna Lloansí Vila on behalf of the PERSEIDA Investigators

Table S1. Characteristics of patients with *RAS* mutations at any time as per liquid biopsy (codon-exon-aminoacid position/ change).

Patient	*RAS* mutant (Exon/Codon/MAF/aminoacid change)			Primary tumor location	Site of metastasis	Days [a]	First-line treatment	Best overall response [b]	PFS (months)
	Baseline	Week 20	Disease progression						
1	*KRAS* 2/13/0.032%/G13D	Wild-type	Wild-type	Left colon	Liver, lung	59	FOLFOX+panitumumab	PR	18.5
2	*KRAS* 2/13/0.046%/ G13D	*KRAS* 2/13/0.059%/ G13D	Wild-type	Left colon	Liver, lung	19	FOLFOX+panitumumab	PR	12.7
3	*KRAS* 2/12/2.952%/G12V	Not Available	no PD	Left colon	Liver	43	FOLFOX+panitumumab	SD	17.4
4	Wild-type	Not Available	*NRAS* 3/61/0.079%/Q61K	Right colon	Peritoneum	512	FOLFOX+panitumumab	PR	13.7
5	*NRAS* 3/61/0.073%/Q61R	Not Available	Wild-type	Left colon	Liver	38	FOLFOX+panitumumab	CR	19.0
6	Wild-type	Wild-type	*KRAS* 2/13/0.083%/ G13D	Left colon	Liver, ganglia, other	25	FOLFOX+cetuximab	PR	8.0
7	*NRAS* 2/12/0.020%/G12D	Wild-type	no PD	Left colon	Ganglia, other	114	Capecitabine	SD	14.5

Patient	*RAS* mutant (Exon/Codon/MAF/aminoacid change)			Primary tumor location	Site of metastasis	Days [a]	First-line treatment	Best overall response [b]	PFS (months)
	Baseline	Week 20	Disease progression						
8	Wild-type	Wild-type	*KRAS* 3/61/0.790%/Q61H	Right colon	Ganglia	41	FOLFOX+panitumumab	PR	19.0
9	*KRAS* 4/146/0.085%/A146T	Wild-type	*KRAS* 4/146/0.041%/A146T	Left colon	Liver, lung, ganglia	528	FOLFOX+panitumumab	PR	7.8
10	*KRAS* 2/12/0.024%/G12C	Not Available	PD, but sample not available	Left colon	Lung, ganglia, other	553	FOLFOX+panitumumab	PD	2.0
11	*KRAS* 2/13/0.021%/G13D	Wild-type	PD, but sample not available	Left colon	Liver, lung	42	FOLFOX+panitumumab	SD	11.4
12	Wild-type	Wild-type	*KRAS* 2/12/0.188%/G12A	Left colon	Lung, ganglia, peritoneum	971	FOLFIRI+cetuximab	SD	11.3
13	*KRAS* 2/12/0.057%/G12D	*KRAS* 2/12/0.397%/G12D	*KRAS* 2/12/0.105%/G12D	Left colon	Ganglia, peritoneum	105	FOLFIRI+panitumumab	SD	5.2
14	*KRAS* 2/12/0.723%/G12R<br>*NRAS* 2/12/0.691%/G12D	*KRAS* 2/12/0.566%/G12R<br>*NRAS* 2/12/0.968%/G12D	PD, but sample not available	Left colon	Liver	13	FOLFOX+panitumumab	CR	6.6
15	*KRAS* 2/13/0.222%/G13D	Wild-type	*KRAS* 2/13/0.214%/G13D	Left colon	Liver	7	FOLFOX+panitumumab	CR	9.3
16	Wild-type	Wild-type	*KRAS* 3 and 2/61 and 12/0.568% and 0.050%/Q61L and G12V<br>*NRAS* 2/61/0.467%/Q61K	Left colon	Liver, lung	13	FOLFOX+panitumumab	PR	15.3
17	*KRAS* 2/13/6.060%/G13D	Not Available	no PD	Left colon	Liver, lung	61	FOLFOX+panitumumab	PR	5.8
18	Wild-type	Wild-type	*NRAS* 3/61/0.221%/Q61K	Left colon	Liver, adrenal	14	FOLFOX+panitumumab	PR	6.9

Patient	*RAS* mutant (Exon/Codon/MAF/aminoacid change)			Primary tumor location	Site of metastasis	Days [a]	First-line treatment	Best overall response [b]	PFS (months)
	Baseline	Week 20	Disease progression						
19	*KRAS* 2/12/0.085%/G12C	Wild-type	*KRAS* 2/12/0.773%/G12C	Left colon	Liver, lung	14	FOLFOX+panitumumab	CR	12.6
20	Wild-type	Wild-type	*KRAS* 2/12/0.042%/ G12C	Left colon	Liver	32	FOLFOX+panitumumab	PR	9.9
21	Wild-type	Wild-type	*KRAS* 2 and 3/12 and 61/0.083% and 0.112%/G12D and Q61H *NRAS* 2/61/0.112%/Q61K	Left colon	Liver	21	FOLFOX+panitumumab	PR	7.1
22	*NRAS* 3/61/0.163%/Q61K	*NRAS* 3/61/0.662%/Q61K	no PD	Left colon	Lung, ganglia	55	FOLFOX+panitumumab	NE	5.3
23	Wild-type	Wild-type	*KRAS* 2/13/0.381%/G13D	Left colon	Liver, ganglia	18	FOLFOX+panitumumab	PR	17.5
24	Wild-type	Wild-type	*NRAS* 2/12/0.341%/G12D	Left colon	Liver	17	FOLFOX+panitumumab	PR	9.8
25	Wild-type	Wild-type	*KRAS* 3/61/9.038%/Q61H	Left colon	Liver, peritoneum	27	5-FU-OX+panitumumab	PR	11.4
26	*KRAS* 2/12/5.951%/G12V	Wild-type	*KRAS* 2/12/0.056%/G12V *NRAS* 2/61/0.056%/Q61R	Left colon	Other	83	FOLFIRI+panitumumab	SD	17.5

a. Days between tissue and sample collection; b. Not confirmed response; Abbreviations: CR; complete response; NE, non-evaluable; PD, progressive disease; PR, partial response; SD, stable disease.

Table S2. Univariable and Multivariable Cox Regression Model for Progression Free Survival (Panitumumab Subpopulation).

A. Univariable Analysis.

Variable	*n* [a]	*p*
RAS status at any time (overall) MAF ≥1%	93	0.230
RAS status at any time (overall) MAF ≥0.1%	93	**0.147**
RAS status at any time (overall) MAF ≥0.02%	93	**0.096**
Number of affected organs (1, >1)	93	0.371
ECOG Performance Status (0, >0)	90	0.294
Age	93	0.382
Localization (Left colon , Right colon)	93	0.242
Primary tumour surgery (Yes, No)	93	0.335
Köhne prognostic score (low risk, intermediate risk, high risk)	83	0.420

Selection of the variables with a *p* value <0.15 for the overall effect of the variable in the univariate Cox regression model. a. A total of 93 patients of the panitumumab subpopulation had available response data.

B. Multivariable Model.

Summary of the number of event and censored values

N obs. read	N obs. used	N event	N censored	Percent censored
93	93	84	9	9.68

Analysis of Maximum Likelihood Estimates

Parameter		Parameter Estimate	Standard Error	Chi-Square	Pr > Chi-Square	Hazard Ratio	95% Hazard Ratio Confidence Limits
RAS status at any time (MAF >0.02)	Mutant vs. Wild-type	0.43120	0.25908	2.7701	0.0960	1.539	0.926–2.557

Only variable *RAS* status at any time (MAF >0.02) was included in the model due to association between variables MAF >0.1% and MAF >0.02% ($p < 0.0001$)

Table S3. Univariable and Multivariable Linear Regression Model for Tumor Burden (Panitumumab Subpopulation).

A. Univariable Analysis.

Effect		Estimate	Standard Error	*t* Value	Pr > |*t*|	Lower	Upper
Intercept		44.67	20.64	2.16	0.0329	3.71	85.64
Liver Metastasis	Yes	29.65	14.43	2.06	0.0425	1.02	58.27
	No	Ref					
cfDNA Baseline		0.0002	0.00005	4.23	<0.0001	0.0001	0.0003
RAS Status Baseline MAF ≥0.02%	Wild-type	12.50	19.57	0.64	0.5243	-26.33	51.34
	Mutant	Ref					

Selection of the variables with a p value < 0.15 for the overall effect of the variable in the univariate linear regression model.

B. Multivariable Model.

Type 3 Tests of Fixed Effects

Effect	F Value	Pr > F
**Liver Metastasis**	4.22	**0.04**
**cfDNA Baseline**	17.91	**<0.0001**
RAS Status Baseline MAF ≥0.02%	0.41	0.52

Least Squares Means

Effect		Estimate	Standard Error	*t* Value	Pr > |*t*|	Lower	Upper
**Liver metastasis**	Yes	95.25	10.65	8.95	<0.0001	74.12	116.38
	No	65.60	14.01	4.68	<0.0001	37.79	93.41

Differences of Least Squares Means

Effect		Estimate	Standard Error	*t* Value	Pr > |*t*|	Lower	Upper
**Liver metastasis**	Yes vs No	29.65	14.43	2.06	0.04	1.02	58.27
